# Sequestration of Martian CO_2_ by mineral carbonation

**DOI:** 10.1038/ncomms3662

**Published:** 2013-10-22

**Authors:** Tim Tomkinson, Martin R. Lee, Darren F. Mark, Caroline L. Smith

**Affiliations:** 1 Scottish Universities Environmental Research Centre, Rankine Avenue, Scottish Enterprise Technology Park, East Kilbride G75 0QF, UK; 2 School of Geographical and Earth Sciences, University of Glasgow, Gregory Building, Lilybank Gardens, Glasgow G12 8QQ, UK; 3 Department of Earth Sciences, Natural History Museum, Cromwell Road, London SW7 5BD, UK; 4 ESA ESTEC, Keplerlaan 1, 200 AG Noordwijk, The Netherlands; 5 UK Space Agency, Atlas Building, Harwell Oxford, Didcot, Oxfordshire OX11 0QX, UK

## Abstract

Carbonation is the water-mediated replacement of silicate minerals, such as olivine, by carbonate, and is commonplace in the Earth’s crust. This reaction can remove significant quantities of CO_2_ from the atmosphere and store it over geological timescales. Here we present the first direct evidence for CO_2_ sequestration and storage on Mars by mineral carbonation. Electron beam imaging and analysis show that olivine and a plagioclase feldspar-rich mesostasis in the Lafayette meteorite have been replaced by carbonate. The susceptibility of olivine to replacement was enhanced by the presence of smectite veins along which CO_2_-rich fluids gained access to grain interiors. Lafayette was partially carbonated during the Amazonian, when liquid water was available intermittently and atmospheric CO_2_ concentrations were close to their present-day values. Earlier in Mars’ history, when the planet had a much thicker atmosphere and an active hydrosphere, carbonation is likely to have been an effective mechanism for sequestration of CO_2_.

## Abstract (web summary)

The mechanism by which Mars lost its early dense and carbon dioxide-rich atmosphere remains relatively unknown. Here, via mineralogical analysis of the Lafayette Martian meteorite, Tomkinson *et al*. infer that carbonation was an effective carbon dioxide sequestration mechanism on an early, water-rich Mars.

## Graphical abstract

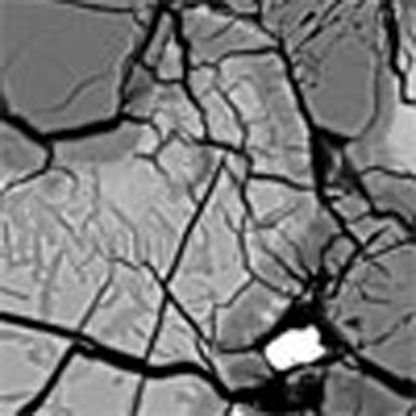

The possible reasons for the depletion of Mars’ early dense and likely CO_2_-rich atmosphere remain contentious^1^,^2^,^3^,^4^,^5^,^6^. On Earth, the replacement of olivine by carbonate, termed carbonation, is an effective way to sequester and store atmospheric CO_2_. For example, the Samail Peridotite in Oman annually binds 4 × 10^7^ kg of CO_2_ via carbonation^7^. The replacement of olivine by carbonate is exothermic, and hence once the activation energy barrier is overcome and while CO_2_-rich fluids and olivine are freely available, the carbonation reaction can be self-perpetuating^8^. On Mars, anhydrous silicate minerals, including olivine, are abundant throughout the crust^9^. Secondary mineral assemblages consisting of carbonates and phyllosilicates have also been observed, exposed at the planet’s surface, by orbiters and rovers^10^,^11^,^12^,^13^,^14^,^15^, and studied directly where they occur in Martian meteorites (for example, the nakhlites)^16^. Their presence is consistent with the interaction of liquid water with the crust, at least sporadically. During the first billion years of Mars’ history, the atmosphere is believed to have been thicker than at present and CO_2_-rich, with up to 5 bars of pressure^1^,^2^,^3^,^4^,^5^,^6^, and dry river valley networks and outflow channels^17^,^18^,^19^ demonstrate the former presence of surface waters. As all the reactants for carbonation (that is, CO_2_, liquid water and olivine) were present on Mars, it has been suggested as a viable mechanism by which the planet lost its early CO_2_-rich atmosphere^20^,^21^.

Here we seek evidence for carbonation by examination of Lafayette, a Martian meteorite that crystallized *c*. 1,300 Ma during the Amazonian epoch. Lafayette is an olivine clinopyroxenite that contains carbonate, which formed by aqueous activity within the outermost *c*. 30 m of the planets’ crust^22^. K-bearing phyllosilicates that are intergrown with the carbonate have been dated to 633±23 Ma (ref. 23), thus temporally constraining the water–rock interaction. Using scanning electron microscopy (SEM), electron probe microanalysis (EPMA) and electron backscatter diffraction (EBSD), we studied a thin section of Lafayette (USNM 1505-5) and grains that had been mechanically separated from bulk samples of the meteorite (NHM 1959 755). The secondary minerals in Lafayette have been identified previously as ferroan saponite, Fe-rich smectite and siderite^24^; (Supplementary Table S1). All three occur within veins that cross-cut olivine grains. Petrographic relationships demonstrate that the siderite formed by isovolumetric replacement of (001) parallel olivine vein walls, which was itself later replaced by Fe-rich smectite. The carbonate has also partly replaced a plagioclase feldspar and apatite-rich mesostasis. Mass balance calculations show that these reactions required only the introduction of liquid water and CO_2_ into the region of the Martian crust from which Lafayette was derived, and that carbonation in one part of the crust may have been coupled with crystallization of the Fe-rich smectite in another. The small volume of siderite in Lafayette indicate that carbonation was limited during the Amazonian, but this reaction is likely to have been far more widespread within crustal rocks that were exposed to groundwater charged with CO_2_ from the thicker Noachian atmosphere.

## Results

### Mineralogy and petrography

Lafayette belongs to the nakhlite group of meteorites that were ejected from Mars at 10.8±0.8 Ma (ref. 25). It is an olivine clinopyroxenite that contains augite (73.5±6.7 vol%) and olivine (16.7±5.7 vol%) with an interstitial groundmass (mesostasis) (9.8±1.2 vol%) that is dominated by plagioclase feldspar with lesser apatite, titanomagnetite and Si-rich glass^24^,^26^,^27^ (Supplementary Table S1). This nakhlite contains a suite of secondary minerals, principally siderite and smectite^24^,^27^, that are identified as Martian in origin^24^; (Supplementary Table S1). The siderite and smectite occur within olivine-hosted veins and form patches between augite and olivine grains. Lafayette has the highest abundance of secondary minerals among the nakhlites, occupying *c*. 1 vol% (ref. 28).

### Olivine-hosted veins

**Figure 1:**
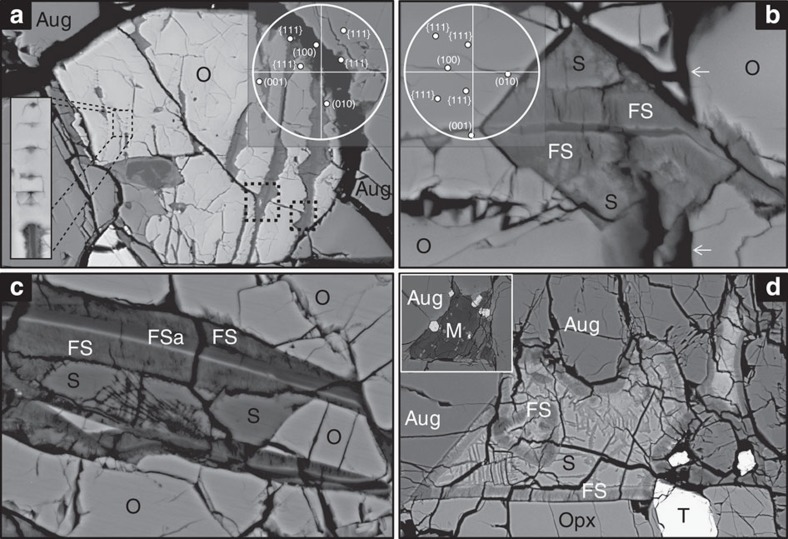
BSE images of Lafayette thin section USNM 1505-5. (**a**) An olivine (O) grain that contains secondary mineral veins (dark grey) and one curving open fracture. The narrow veins extend from the interface with augite (Aug) and are discontinuous. The lower left hand side inset highlights a discontinuous vein. Wider veins cross-cut the entire grain and have coarsely serrated walls. They contain an axial strip of ferrous saponite that is flanked by Fe-rich smectite. The two dotted black squares indicate where veins cross-cut the open fracture. The inset EBSD pole figure (top right) shows that the veins lie parallel to the trace of (001)_ol_. Image width = 242 μm. (**b**) An olivine-hosted secondary mineral vein that cross-cuts a vertical open fracture (arrows). The axis of the vein comprises ferrous saponite (dark grey). In the centre of the field of view is a notch that contains Fe-rich smectite (FS) and siderite (S). The inset EBSD pole figure (top left) shows that the axis of the vein is close to the trace of (001)_ol_ and the walls of the notches lie parallel to {111}_ol_. Image width = 49 μm. (**c**) Two olivine-hosted secondary mineral veins. Both veins contain axial ferrous saponite (FSa) that has a Fe-rich rim (white) and is flanked by bands of Fe-rich smectite (FS). Between the two veins is a ~10-μm wide selvage of olivine (O) plus siderite (S). The siderite contains a spray of phyllosilicate fibres. Image width = 52 μm. (**d**) A patch of siderite (S) and Fe-rich smectite (FS) that is enclosed by augite (Aug), orthopyroxene (Opx) and titanomagnetite (T). Fe-rich smectite occurs around the margins of the patch and also cross-cuts the siderite. The inset shows an unaltered patch of mesostasis (M) that is comprised mainly of plagioclase feldspar with some titanomagnetite crystals (white). The insert image width = 198 μm. The size, shape and petrographic context of the mesostasis is very similar to the patch of siderite plus Fe-rich smectite. Image width = 202 μm.

EBSD mapping of olivine grains demonstrates that the axes of most of the veins lie parallel to (001)_ol_ (Fig. 1a). Two vein types can be recognized by differences in their size and mineralogy: the narrow veins are 1–2 μm wide, have planar or finely serrated walls and contain a compact and very finely crystalline Mg-Fe silicate that has been identified as ferrous saponite^24^; (Table 1; Supplementary Table S1). Many of the narrow veins extend only part way into olivine grains from intergranular boundaries or from intragranular fractures (Fig. 1a). These veins pass into lines of ferrous saponite inclusions whose faceted shape is defined by {111}_ol_ (Fig. 1a). In contrast, the larger veins are up to 40 μm wide, cross-cut entire olivine grains and have coarsely serrated walls (Fig. 1a). Some veins originate from intragranular fractures, whereas others cross-cut the fractures and so clearly post-date them (Fig. 1a,b). These veins contain a 1–2-μm-wide axial strip of ferrous saponite that is flanked by bands of a fibrous Mg-Fe phyllosilicate up to 2 μm wide (Fig. 1b,c). This phyllosilicate has been previously interpreted to be a Fe-rich smectite^24^ (or a smectite intergrown with serpentine^28^); (Table 1). Siderite (Table 1) occurs only within those parts of veins that are wider than 4 μm, which corresponds to the deepest notches (Fig. 1b). The walls of these notches lie parallel to the traces of {102}_ol_ or {111}_ol_ (Fig. 1b). Siderite has an irregular interface with the Fe-rich smectite (Fig. 1c) and is also cross-cut by narrow smectite veins and occasionally also contains smectite ‘sprays’ and ‘rosettes’^24^,^28^. Where veins bifurcate, a wedge composed of olivine, siderite, Fe-rich smectite or any combination of these minerals occurs between them (Fig. 1c).

### Mesostasis patches

Patches containing siderite, Fe-rich smectite and titanomagnetite are 100–150 μm in size (Fig. 1d) and restricted in their occurrence to discrete millimetre-sized regions of Lafayette. These patches are comparable in size, shape and petrographic context to pristine areas of mesostasis (Fig. 1d). The mesostasis siderite is depleted in Mn and enriched in Ca relative to that in the olivine-hosted veins (Table 1). In all occurrences, the mesostasis siderite is enclosed and cross-cut by Fe-rich smectite (Fig. 1d).

## Discussion

**Figure 2:**
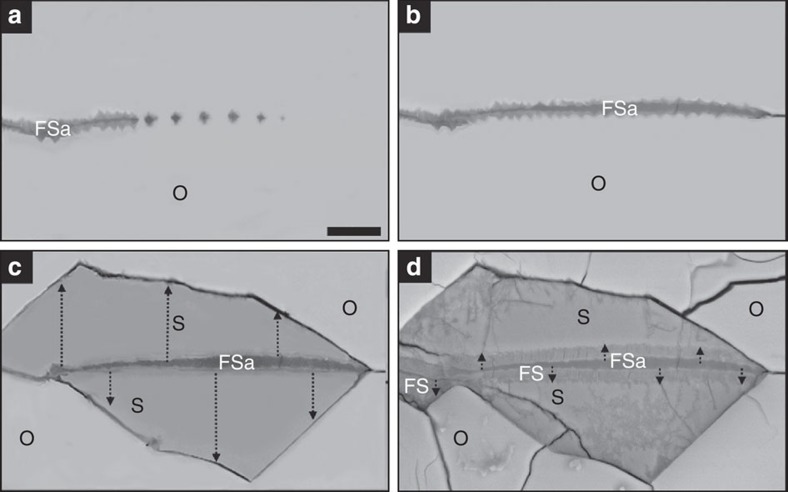
The sequence of replacement reactions within an olivine-hosted vein. (**a**–**c**) are cartoons made by editing the BSE image in **d**. (**a**) Propagation of a ferrous saponite (FSa) vein through an olivine (O) grain parallel to (001). (**b**) Ferrous saponite inclusions have merged to make a continuous vein, but it has stopped short of the right hand side of the olivine grain. (**c**) Crystallographically controlled replacement of the olivine vein walls by siderite (S). (**d**) Replacement of siderite by Fe-rich smectite (FS), working inwards from its interface with ferrous saponite, is the final event that is recorded. Grain separated from NHM 1959 755. Scale bar=10μm.

The olivine-hosted veins of ferrous saponite are interpreted to be the first products of water–rock interaction. The grain boundaries and intragranular fractures from which the veins have propagated must have served as conduits for the aqueous solutions. As the fine-scale serrations on vein walls are comparable in morphology to etch pits in olivine grains from the Nakhla (Martian) meteorite^29^, and naturally weathered terrestrial rocks^30^. The veins are inferred to have formed by dissolution of olivine and concomitant precipitation of ferrous saponite (Fig. 2a,b; Supplementary Note 1; Supplementary Table S2). The veins are formed by coalescence of lines of ferrous saponite inclusions beyond their tips, and they have probably exploited defects parallel to (001)_ol_, such as subgrain boundaries. This mechanism of vein formation is equivalent to ‘centripetal’ replacement of terrestrial olivine, and serrated vein walls are also a characteristic of this reaction^31^. The presence of Na, Al, P, K and Ca in the ferrous saponite^24^ (Supplementary Note 1) indicates that the cations were not sourced solely from the olivine, and are likely to have been derived from dissolution of mesostasis feldspar and apatite.

The formation of ferrous saponite veins was an important driver of subsequent olivine carbonation for two reasons. First, the vein walls served as conduits for CO_2_-rich fluids to gain access to grain interiors, and it has been demonstrated experimentally that partial serpentinisation of terrestrial olivine increases its susceptibility to carbonation^32^. Secondly, the absence of siderite on grain boundaries that lie parallel to (010)_ol_ and (100)_ol_ (Fig. 1a) shows that replacement was crystallographically controlled and most effective on surfaces parallel to (001)_ol_ (that is, the vein walls). This control on siderite formation by the crystal structure of olivine demonstrates that the carbonate has formed by replacement. Additional evidence for replacement is that siderite cross-cuts pre-existing fractures (Fig. 1a,b), and is intergrown with wedges of olivine between closely spaced veins (Fig. 1c). Siderite grew most rapidly parallel to [001]_ol_ and the dissolution–reprecipitation front was guided by the olivine crystal structure to make the {102}/{111} notches (Fig. 2c). Such coarsely serrated olivine–carbonate interfaces are also diagnostic of terrestrial carbonation^7^,^32^. The olivine-hosted siderite was subsequently replaced by the Fe-rich smectite^28^ on a volume-for-volume basis. The dissolution–reprecipitation front extended uniformly inwards from the ferrous saponite–siderite interface so that only carbonate in the deepest notches remains (Figs 1b and 2d).

The patches of siderite and Fe-rich smectite between augite and olivine grains are also interpreted to have formed by isovolumetric replacement, first of the mesostasis minerals and second of siderite by Fe-rich smectite. The presence of mesostasis-derived elements in the ferrous saponite indicates that the apatite and plagioclase feldspar had undergone dissolution during early stages of water–rock interaction. However, two lines of evidence demonstrate that the siderite formed predominantly by replacement rather than by filling pores resulting from the congruent dissolution of the mesostasis: (i) nowhere in Lafayette has siderite been observed to cement fractures, despite the evidence that they were present before carbonation (Fig. 1a,b), and (ii) the mesostasis siderite is enriched in Ca and depleted in Mn relative to olivine-hosted siderite, which mirrors the compositions of the precursors (that is, olivine is the main source of Mn in Lafayette and mesostasis is the main source of Ca; see below). The mesostasis siderite was subsequently replaced by Fe-rich smectite (Fig. 1d), which is compositionally comparable to Fe-rich smectite in the olivine-hosted veins (Table 1). Differences within Lafayette in the degree of replacement of mesostasis may reflect contrasts in original mineralogy that rendered some regions especially susceptible to carbonation (for example, greater volume of Si-rich glass or apatite). However, in the absence of evidence for significant millimetre-scale heterogeneities in the mineralogy of the mesostasis, it is more likely that the carbonating aqueous solutions were in contact with some parts of Lafayette for long periods of time. This is most likely because of the presence of localized regions of elevated permeability, for example, resulting from the partial dissolution of the mesostasis before or during ferrous saponite formation.

The conclusion that siderite and Fe-rich smectite both formed by isovolumetric replacement can be tested by calculating the exchange of elements during these reactions. These calculations assume that only water and CO_2_ were sourced from outside of Lafayette. Carbonation of olivine required import to the reaction site of Ca, Mn and CO_2_ if cations common to both minerals had been conserved (Supplementary Note 1 and Supplementary Table S3), and replacement of the plagioclase-apatite mesostasis by siderite necessitated the additional introduction of Mg and Fe (Supplementary Note 1 and Supplementary Table S4). The olivine-hosted siderite is Mg-poor despite the abundance of Mg in the precursor silicate (Table 1). However, crystallization of Ca-carbonates is often kinetically favoured over Mg-carbonates during replacement of terrestrial olivine owing to the weaker hydration of the Ca over the Mg ions^32^. As carbonation of olivine would have enriched the parent fluid with respect to Mg and Fe, it may have been coupled with replacement of the mesostatis by siderite (Supplementary Note 1 and Supplementary Tables S3 and S4). These reactions do not balance precisely owing to a small deficit of Ca and Mn. The Mn may have been sourced from earlier replacement of olivine by ferrous saponite (Supplementary Note 1 and Supplementary Table S2), and the Ca could have come from congruent dissolution of the mesostasis. As siderite obtained a maximum of 53% (by mass) of its cations from olivine and 22% (by mass) from the mesostasis (Supplementary Tables S3 and S4), supply of elements from the dissolving primary minerals may have been insufficient to have supersaturated the interfacial solutions with respect to siderite. The more significant driver for carbonation is likely to have been an increase in the pH (from acidic to alkaline) and bicarbonate activity of the fluid films accompanying dissolution of olivine and the mesostasis.

The carbonation reactions and subsequent replacement of the siderite by Fe-rich smectite may also have been coupled. All of the ions required for the Fe-rich smectite entered the bulk solution during carbonation of olivine and mesostasis, or were acquired from the siderite during its replacement (Supplementary Note 1 and Supplementary Tables S3–S6). Linked crystallization of siderite and Fe-rich smectite in Lafayette is expected because these complementary reactions are commonplace during the experimental and natural carbonation of olivine^7^,^33^. Replacement of siderite by Fe-rich smectite would have enriched bulk solutions in Ca, Mn, Fe and CO_2_ that could have been available for further carbonation (Supplementary Note 1 and Supplementary Tables S5 and S6). Therefore, depending on the scale and interconnectivity of the aqueous system, siderite and Fe-rich smectite could have been crystallizing simultaneously within different parts of the nakhlite parent rock, with isovolumetric carbonation in one region stimulating crystallization of Fe-rich smectite in another. This suggestion is consistent with previous calculations of element exchange during aqueous alteration of Lafayette^24^, which indicated that water/rock ratios were low, and most of the secondary mineral cations were derived locally from olivine and the mesostasis.

A previous model for formation of secondary minerals within the nakhlite meteorites^28^ hypothesized that they were aqueously altered within a post-impact hydrothermal system, with the siderite cementing serrated fractures that had been opened by shock. The present study has demonstrated the coarsely serrated veins in Lafayette formed by crystallographically controlled carbonation of olivine, so there is no evidence (or requirement) for a genetic link between aqueous alteration of Lafayette and an impact event.

On Mars, carbonate minerals are potentially important sinks for CO_2_ with the ability to store the gas over geological timescales. The absence of pore-filling siderite in Lafayette shows that within the region of the crust of Mars that this meteorite has sampled, carbon has been mineralized by replacement. Secondary minerals occupy 9 vol% of each Lafayette olivine grain^28^, and as siderite once occupied two thirds of each vein (that is, the current volume of siderite plus Fe-rich smectite), Lafayette originally contained 1 vol% of olivine-hosted siderite, corresponding to storage of 15.88 kg of CO_2_ m^−3^.

**Figure 3:**
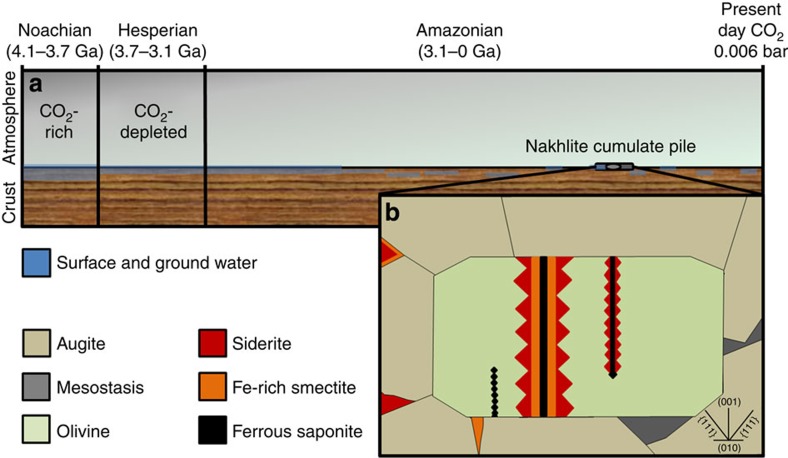
Mars’ H_2_O and CO_2_ reservoirs and Amazonian secondary mineral formation. (**a**) Summary of the relative concentrations of atmospheric CO_2_ over Mars’ history, and the relative abundances of surface and groundwater. (**b**) Diagram showing the distribution of primary and secondary minerals in Lafayette. The olivine grain is shown cut parallel to (100), and the orientation of various planes within the grain is shown in the lower right hand side. The image (**b**) width is ~250 μm.

Given the evidence for CO_2_ drawdown in the Amazonian, we hypothesized that carbonation was a major CO_2_ sink during the Noachian with prevailing environmental conditions enhancing the effectiveness of this reaction (that is, higher atmospheric CO_2_ concentrations^1^,^2^,^3^,^4^,^5^,^6^ and greater availability of liquid water^17^,^18^,^19^; Fig. 3). For example, complete carbonation of olivine and mesostasis in a Noachian Lafayette-type crust would store 312–513 kg CO_2_ m^−3^ (175–356 kg CO_2_ m^−3^ in olivine and 137–175 kg CO_2_ m^−3^ in the mesostasis). However, the ALH 84001 orthopyroxenite meteorite is currently our only physical sample of Noachian crust and relative to Lafayette, its equivalent carbonation potential is much lower (that is, it has only minor quantities of olivine^34^ and ~2% maskelynite^35^). As such, it is difficult to quantify the global extent and impact of carbonation with respect to the evolution of the Martian atmosphere. However, remote sensing data from the Noachian Nili Fossae region of Mars^13^,^20^,^21^ that show the intimate association of olivine with carbonate support a model of CO_2_ sequestration by mineral carbonation.

## Methods

### Petrography

This study used Lafayette thin section USNM 1505-5 and olivine grains that had been mechanically separated from the bulk sample NHM 1959 755, which were embedded in resin and polished. Following carbon coating, backscattered electron (BSE) images were obtained using a Zeiss Sigma field-emission SEM operated at 20 kV/1 nA. The crystallographic orientation of secondary mineral veins within olivine grains was determined by EBSD using a FEI Quanta 200F field-emission SEM equipped with a TSL-EDAX EBSD system. EBSD mapping was undertaken following removal of the carbon coat and with the microscope operated at low vacuum (~50 Pa) and 20 kV. Maps were acquired at a rate of ~20 Kikuchi patterns per seconds and with a step size of ~0.1 μm. The orientations of poles to various planes are plotted in upper hemisphere stereographic pole figures. Kikuchi patterns could not be obtained from the siderite.

### Quantitative chemical analysis

Analyses of olivine were acquired using a Cameca SX00 electron probe at the University of Edinburgh. Calibration used jadeite (Na), spinel (Mg, Al), forsterite (Si), apatite (P), barite (S), orthoclase (K), wollastonite (Ca), rutile (Ti), Cr metal (Cr), Mn metal (Mn) and fayalite (Fe). Na, Mg, Si and Fe were analysed at 15 kV/10 nA, with peak/background count times of between 20/10 and 40/20 s., whereas Al, P, S, K, Ca, Ti, Cr and Mn were analysed at 15 kV/100 nA, with peak/background count times of between 20/10 and 60/30 s. All analyses were obtained in spot mode. Detection limits were 0.01 wt% Na, Mg, and K, 0.03 wt% Al, Ca and Ti, 0.02 wt% Si, P and S, 0.10 wt% Cr, 0.05 wt% Mn and 0.11 wt% Fe. P and Ti were undetectable in all analyses. The other minerals were chemically analysed at the University of Glasgow using a Zeiss Sigma analytical SEM equipped with an Oxford Instruments X-Max silicon drift energy dispersive X-ray detector. Operating conditions were 15 kV/1.1 nA, and analyses were obtained by rastering the electron beam over ~2 × 2 μm areas for 60 s. Calibration used jadeite (Na), periclase (Mg), corundum (Al), rhodonite (Si, Mn), apatite (P), celestite (S), halite (Cl), orthoclase (K), calcite (Ca), rutile (Ti) and garnet (Fe). All of these elements were sought in phyllosilicate analyses, whereas only Mg, Ca, Mn, Fe and Sr were analysed for in the siderite. Detection limits were 0.09 wt% Na, 0.05 wt% Mg and Ni, 0.10 wt% Al, P, S and Cl, 0.09 wt% Si, 0.13 wt% K, 0.15 wt% Ca, 0.20 wt% Ti, 0.40 wt% Mn, 0.30 wt% Fe, and 0.14 wt% Sr. P, Cl and Ti were undetectable in all analyses.

## Author contributions

D.F.M. and M.R.L. project conception. T.T. prepared the samples and together with M.R.L. undertook the electron beam imaging and analysis, and produced the figures and tables. M.R.L. performed the mass balance calculations. All the authors contributed to the interpretations and writing of the paper.

## Additional information

**How to cite this article:** Tomkinson, T. *et al*. Sequestration of Martian CO_2_ by mineral carbonation. *Nat. Commun.* 4:2662 doi: 10.1038/ncomms3662 (2013).

## Supplementary Material

Supplementary Information Supplementary Tables S1-S6, Supplementary Note 1 and Supplementary References

## Figures and Tables

**Table 1:** Chemical compositions of minerals involved in the replacement reactions.

	**Olivine**	**Olivine-hosted Fe-rich smectite**	**Olivine-hosted siderite**	**Plagioclase feldspar**	**Mesostasis Fe-rich smectite**	**Mesostasis siderite**
SiO_2_	31.82 (0.35)	38.01 (4.91)	n.a.	59.43	38.85 (1.50)	n.a.
Al_2_O_3_	d.l.	3.10 (0.80)	n.a.	24.12	3.47 (0.31)	n.a.
FeO	51.70 (0.59)	30.20 (3.66)	29.11 (6.65)	0.78	31.66 (1.86)	38.55 (0.79)
MnO	1.06 (0.11)	1.71 (0.75)	14.95 (5.77)	d.l.	0.93 (0.12)	4.21 (1.52)
MgO	14.23 (0.50)	9.72 (1.58)	0.15 (0.11)	0.07	9.76 (0.49)	0.36 (0.21)
CaO	0.20 (0.03)	1.50 (0.27)	15.32 (0.98)	6.91	1.66 (0.18)	16.64 (0.85)
Na_2_O	d.l.	0.40 (0.03)	n.a.	7.06	0.35 (0.07)	n.a.
K_2_O	d.l.	0.59 (0.20)	n.a.	0.68	0.65 (0.19)	n.a.
SO_3_	d.l.	0.10 (0.05)	n.a.	d.l.	0.19 (0.07)	n.a.
H_2_O	-----	14.67	-----	-----	12.48	-----
CO_2_	-----	-----	40.47	-----	-----	40.24
Total	99.01	100.00	100.00	99.05	100.00	100.00
*n*	35	4	18	1	7	8
						
	Ions per 4 O	Ions per 16 O*	Ions per 3 O	Ions per 8 O	Ions per 16 O*	Ions per 3 O
Si	0.985	3.490	-----	2.674	3.651	-----
Al	0.000	0.335	-----	1.279	0.384	-----
Fe	1.338	2.319	0.445	0.029	2.488	0.589
Mn	0.028	0.133	0.231	-----	0.074	0.065
Mg	0.656	1.331	0.004	0.005	1.367	0.010
Ca	0.007	0.148	0.300	0.333	0.167	0.326
Na	-----	0.071	-----	0.615	0.064	-----
K	0.000	0.069	-----	0.039	0.078	-----
S	-----	0.007	-----	-----	0.013	-----
H	-----	8.992	-----	-----	7.829	-----
C	-----	-----	1.010	-----	-----	1.005
	Fa=67.1		Cc=30.6	Ab=62.3		Cc=32.9
	Fo=32.9		Mg=0.4	An=33.7		Mg=1.0
			Sd=45.4	Or=4.0		Sd=59.5
			Rd=23.6			Rd=6.6

d.l., below detection limits;. n.a., not analysed for.

Figures in parentheses are standard deviations. H_2_O and CO_2_ calculated by difference.

^*^The phyllosilicates contain intergrowths of different minerals and so will inevitably deviate from ideal stoichiometry.
