# Supplementary material for: Sequestration of Martian CO2 by mineral carbonation
Source: Nat Commun. 2013 Oct 22;4:2662. doi: 10.1038/ncomms3662 (PMC4354006; doi:10.1038/ncomms3662)
Supplement: Supplementary Information — Supplementary Tables S1-S6, Supplementary Note 1 and Supplementary References [file ncomms3662-s1.pdf]

Supplementary Information

Supplementary Tables

Supplementary Table S1. Minerals identified in the Lafayette meteorite

Mineral	Technique	Reference
Apatite	EPMA	36
Augite	EPMA	37, 38, 39, 24, 27 & This study
Olivine	EPMA	37, 40, 39, 26, 27 & This study
Plagioclase feldspar	EPMA	37, 41 & This study
	Raman spectroscopy	41
Ferrous saponite	TEM	24 & 42
Fe-rich smectite	EPMA	42 & 28
	TEM	24, & 28
	Quantitative EDX	24 & This study
	Raman spectroscopy	42,
Siderite	EPMA	43 & 44
	Quantitative EDX	This study
	Raman spectroscopy	43

EMPA denotes electron microprobe analysis, TEM = Transmission electron microscopy and EDX = energy-dispersive X-ray spectrometer.

Supplementary Table S2. Replacement of 100 cm^3 of olivine by ferrous saponite via the reaction in Supplementary Note 1

	In olivine (g)	Added from solution (g)	In ferrous saponite(g)	Lost to solution (g)
H	-----	1.0	1.0	-----
O	138.9	8.0	104.0	42.9
Na	-----	0.9	0.9	-----
Mg	34.8	-----	19.8	15.1
Al	-----	4.3	4.3	-----
Si	60.3	-----	49.9	10.4
P	-----	0.3	0.3	-----
K	-----	1.2	1.2	-----
Ca	0.9	-----	0.9	-----
Mn	3.6	-----	0.5	3.3
Fe	162.4	-----	46.4	116.0
Total	400.9	15.7	229.2	187.7

Supplementary Table S3. Carbonation of 100 cm^3 of olivine *via* the reaction in Supplementary Note 1

	In olivine (g)	Added from solution (g)	In siderite (g)	Lost to solution (g)
CO_2	-----	158.8	158.8	-----
O	138.9	-----	57.8	81.1
Si	60.3	-----	-----	60.1
Mg	34.8	-----	-----	34.8
Ca	0.9	42.9	43.4	-----
Mn	3.6	42.3	45.6	-----
Fe	162.4	-----	90.7	72.6
Total	400.9	244.0	396.3	248.6

Siderite inherited a maximum of 53% (by mass) of its cations from olivine.

Supplementary Table S4. Isovolumetric carbonation of the plagioclase and apatite via the reaction in Supplementary Note 1

	In plagioclase and apatite (g)	Added from solution (g)	In siderite (g)	Lost to solution (g)
CO_2	-----	158.8	158.8	
O	122.9	-----	57.8	65.6
Na	12.4	-----	-----	12.4
Mg	0.2	0.7	0.9	
Al	30.0	-----	-----	30.0
Si	65.3	-----	-----	65.3
P	5.5	-----	-----	5.5
Cl	1.1	-----	-----	1.1
K	1.4	-----	-----	1.4
Ca	23.4	24.4	47.8	-----
Mn	-----	13.9	13.9	-----
Fe	16.2	102.7	118.9	-----
Total	278.4	300.5	398.1	181.3

Siderite inherited a maximum of 22% (by mass) of its cations from the mesostasis.

Supplementary Table S5. Isovolumetric replacement of 100 cm^3 olivine-hosted siderite by Fe-rich smectite via the reaction in Supplementary Note 1

	In siderite (g)	Added from solution (g)	In Fe-rich smectite (g)	Lost to solution (g)
H	-----	6.2	3.8	2.4
CO_2	158.8	-----	-----	158.8
O	57.8	88.3	107.5	38.7
Na	-----	0.7	0.7	-----
Al	-----	3.8	3.9	-----
Si	-----	41.0	41.2	-----
S	-----	0.1	0.1	-----
K	-----	1.2	1.1	-----
Mg	-----	13.1	13.3	-----
Ca	43.4	-----	2.5	40.9
Mn	45.6	-----	3.0	42.6
Fe	90.7	-----	54.4	36.3
Total	396.3	154.4	231.5	319.7

Supplementary Table S6. Isovolumetric replacement of 100 cm^3 of mesostasis siderite by Fe-rich smectite via the reaction in Supplementary Note 1

	In siderite (g)	Added from solution (g)	In Fe-rich smectite (g)	Lost to solution (g)
H	-----	5.8	3.2	2.6
CO_2	158.8	-----	-----	158.8
O	57.8	68.8	104.4	20.8
Na	-----	0.6	0.6	-----
Al	-----	4.2	4.2	-----
Si	-----	42.0	42.0	-----
S	-----	0.1	0.1	-----
K	-----	1.3	1.3	-----
Mg	0.9	12.8	13.7	-----
Ca	47.8	-----	2.9	44.9
Mn	13.9	-----	1.6	12.3
Fe	118.9	-----	57.0	61.9
Total	398.1	135.6	231.0	301.3

Supplementary Note 1

Element exchange accompanying isovolumetric replacement of 100 cm^3 of olivine by ferrous saponite.

$$2.17Mg_{0.66}Ca_{0.01}Mn_{0.03}Fe_{1.34}Si_{0.99}O_4 + 0.16Al + 0.03K + 0.04Na + 0.01P + 0.5H_2O \rightarrow 0.5(Fe^{2+}_{1.66}Mg_{1.63}Mn_{0.02}Ca_{0.04}K_{0.06}Na_{0.08}P_{0.01})(Si_{3.55}Al_{0.31})O_{11}(OH)_2 + 0.37Si + 0.62Mg + 2.08Fe + 0.06Mn + 2.68O$$

Element exchange accompanying isovolumetric replacement of 100 cm^3 of olivine by siderite.

$$2.17Mg_{0.66}Ca_{0.01}Mn_{0.03}Fe_{1.34}Si_{0.99}O_4 + 0.77Mn + 1.07Ca + 3.61CO_2 \rightarrow 3.61Ca_{0.30}Mn_{0.23}Fe_{0.45}CO_3 + 2.14Si + 1.43Mg + 1.30Fe + 5.07O$$

Element exchange accompanying isovolumetric replacement of the mesostasis (assumed to comprise 90 cm^3 of plagioclase feldspar and 10 cm^3 of apatite) by siderite.

$$0.87K_{0.04}Na_{0.62}Ca_{0.33}Mg_{0.01}Fe_{0.03}Si_{2.67}Al_{1.28}O_8 + 0.06Ca_{4.93}Si_{0.04}Fe_{0.05}(PO_4)_{2.97}(F_{0.04}Cl_{0.53}H_{0.03}O_{0.15}) + 0.03Mg + 0.61Ca + 0.25Mn + 1.8Fe + 3.61CO_2 \rightarrow 3.61Mg_{0.01}Ca_{0.33}Mn_{0.07}Fe_{0.59}CO_3 + 2.33Si + 1.12Al + 0.54Na + 0.03K + 0.18P + 0.03Cl + 4.1O$$

Element exchange accompanying isovolumetric replacement of 100 cm^3 of olivine-hosted siderite by Fe-rich smectite.

$$3.61Ca_{0.30}Mn_{0.23}Fe_{0.45}CO_3 + 1.46Si + 0.54Mg + 0.14Al + 0.01S + 0.03K + 0.03Na + 3.10H_2O + 2.42O \rightarrow 0.42Ca_{0.15}Na_{0.07}K_{0.07}S_{0.01}Mg_{1.30}Mn_{0.13}Fe^{2+}_{2.32}Si_{3.49}Al_{0.34}(OH)_2\cdot H_{6.99}O_{14} + 3.61CO_2 + 0.65Fe + 1.02Ca + 0.78Mn + 1.21H_2O$$

Element exchange accompanying isovolumetric replacement of 100 cm^3 of mesostasis siderite by Fe-rich smectite.

$$3.61Mg_{0.01}Ca_{0.33}Mn_{0.07}Fe_{0.59}CO_3 + 1.50Si + 0.16Al + 0.03K + 0.03Na + 0.53Mg + 0.01S + 2.90H_2O + 1.3O \rightarrow 0.41Ca_{0.17}Na_{0.06}K_{0.08}S_{0.01}Mg_{1.30}Mn_{0.07}Fe^{2+}_{2.49}Si_{3.65}Al_{0.38}(OH)_2\cdot H_{5.83}O_{14} + 3.61CO_2 + 0.01S + 1.11Fe + 1.12Ca + 0.22Mn + 1.3H_2O$$

Supplementary References

36. McCubbin, F. M., Elardo, S. M., Shearer, C. K., Smirnov, A., Hauri, E. H. & Draper, D. S. A petrogenetic model for the comagmatic origin of the chassignites and nakhlites: Inferences from chlorine-rich minerals, petrology, and geochemistry. *Meteorit. Planet. Sci.* **48**, 819–853 (2013).

37. Bunch T.E. & Reid A.M. The Nakhlites, I. Petrography and mineral chemistry. *Meteoritics* **10**, 303-315 (1975).

38. Berkley, J. L., Keil, K. & Prinz, M. Comparative petrology and origin of Governador Valadares and other nakhlites. *11th Lunar and Planetary Science Conference, Houston, Texas*, USA #1089–1102 (1980).

39. Harvey, R. P. & McSween, H. Y. Petrogenesis of the Nakhlite meteorites: Evidence from cumulate mineral zoning. *Geochim. Cosmochim. Acta*. **56**, 1655-1663 (1992).

40. Smith J. V., Steele I. M. & Leitch C. A. Mineral chemistry of the Shergottites, Nakhlites, Chassigny, Brachina, Pallisites and Ureilites. *14th Lunar and Planetary Science Conference, Houston, Texas, USA* #B229-236 (1983).

41. Fritz, J., Greshake, A. & Stöffler, D. Micro-Raman spectroscopy of plagioclase and maskelynite in Martian meteorites: Evidence of progressive shock metamorphism. *Antarct. Meteorite Res.* **18**, 96-116 (2005).

42. Kuebler, K., Jolliff, B.L., Wang, A. & Haskin L.A. A survey of olivine alteration products using Raman spectroscopy. *35th Lunar and Planetary Science Conference, Houston, Texas*, USA #1704 (2004).

43. Vicenzi, E. P., Tobin, K., Heaney, P.J., Onstott , T.C. & Chun J. Carbonate in Lafayette meteorite: A detailed microanalytical study. *60th Meteoritical. Society Conference*. #5292 (1997).

44. Bridges, J. C. & Grady M. M. Evaporite mineral assemblages in the nakhlite (martian) meteorites. *Earth Planet. Sci. Lett.* **176**, 267–279 (2000).
